# Guideline-based quality assurance: a conceptual framework for the definition of key elements

**DOI:** 10.1186/s12913-021-06148-2

**Published:** 2021-02-24

**Authors:** Elena Parmelli, Miranda Langendam, Thomas Piggott, Jan Adolfsson, Elie A. Akl, David Armstrong, Jeffrey Braithwaite, Romina Brignardello-Petersen, Markus Follmann, Zbigniew Leś, Joerg J. Meerpohl, Luciana Neamtiu, Amir Qaseem, Paolo Giorgi Rossi, Zuleika Saz-Parkinson, Philip J. van der Wees, Holger J. Schünemann

**Affiliations:** 1 European Commission, Joint Research Centre (JRC), Via E. Fermi 2749 – TP 127, I-21027 Ispra, Italy; 2 Department of Epidemiology and Data Science, Amsterdam UMC, Amsterdam Public Health institute, University of Amsterdam, Amsterdam, Netherlands; 3 Department of Health Research Methods, Evidence, and Impact McMaster University Health Sciences Centre, Room 2C16, 1280 Main Street, West Hamilton, ON L8N 4K1 Canada; 4 Swedish Agency for Health Technology Assessment and Assessment of Social Services, Sweden & The Department of Clinical Science, Intervention and Technology, Karolinska Institutet, Stockholm, Sweden; 5 American University of Beirut, Beirut, Lebanon; 6 Farncombe Family Digestive Health Research Institute, McMaster University, Hamilton, Canada; 7 Department of Medicine, McMaster University, Hamilton, ON Canada; 8 Australian Institute of Health Innovation, Macquarie University, Sydney, Australia; 9 German Cancer Society, Berlin, Germany; 10 Evidence Prime, Kraków, Poland; 11 Institute for Evidence in Medicine, Medical Center – University of Freiburg, Faculty of Medicine, University of Freiburg, Freiburg, Germany; 12 Cochrane Germany, Cochrane Germany Foundation, Freiburg, Germany; 13 American College of Physicians, Philadelphia, PA USA; 14 Epidemiology Unit, Azienda USL - IRCCS di Reggio Emilia, Reggio Emilia, Italy; 15 Department of Rehabilitation and IQ Healthcare, Radboud University Medical Center, Radboud Institute for Health Sciences, Nijmegen, Netherlands

**Keywords:** Guidelines, Quality indicators, Healthcare quality, Quality assurance, Recommendations

## Abstract

**Background:**

In 2017, the European Commission’s Joint Research Centre (JRC) started developing a methodological framework for a guideline-based quality assurance (QA) scheme to improve cancer quality of care. During the first phase of the work, inconsistency emerged about the use of terminology for the definition, the conceptual underpinnings and the way QA relates to health questions that are answered in guidelines.

The objective of this final of three articles is to propose a conceptual framework for an integrated approach to guideline and QA development and clarify terms and definitions for key elements. This work will inform the upcoming European Commission Initiative on Colorectal Cancer (ECICC).

**Methods:**

A multidisciplinary group of 23 experts from key organizations in the fields of guideline development, performance measurement and quality assurance participated in a mixed method approach including face-to-face dialogue and several rounds of virtual meetings. Informed by results of a systematic literature review that indicated absence of an existing framework and practical examples, we first identified the relations of key elements in guideline-based QA and then developed appropriate concepts and terminology to provide guidance.

**Results:**

Our framework connects the three key concepts of quality indicators, performance measures and performance indicators integrated with guideline development. Quality indicators are constructs used as a guide to monitor, evaluate, and improve the quality of the structure, process and outcomes of healthcare services; performance measures are tools that quantify or describe measurable elements of practice performance; and performance indicators are quantifiable and measurable units or scores of practice, which should be guided by guideline recommendations.

**Conclusions:**

The inconsistency in the way key terms of QA are used and defined has confused the field. Our conceptual framework defines the role, meaning and interactions of the key elements for improving quality in healthcare. It directly builds on the questions asked in guidelines and answered through recommendations. These findings will be applied in the forthcoming ECICC and for the future updates of ECIBC. These are large-scale integrated projects aimed at improving healthcare quality across Europe through the development of guideline-based QA schemes; this will help in implementing and improving our approach.

## Background

In its effort to improve cancer care, the European Commission Joint Research Centre (JRC) has been responsible for the scientific and technical coordination of the European Commission Initiative on Breast Cancer (ECIBC - https://healthcare-quality.jrc.ec.europa.eu/). ECIBC is a person-centred initiative, involving 35 participating countries. The two main objectives of the project are: 1. the development of evidence-based breast cancer guidelines on screening and diagnosis; and 2. the development of a quality assurance (QA) scheme for breast cancer services [1, 2]. The JRC involved two multidisciplinary and independent panels working simultaneously on the ECIBC guidelines and on the corresponding Quality Assurance (QA) scheme.

In ECIBC, QA is defined as the part of quality management which is directed at the creation of trust that quality requirements are satisfied (ISO 9000:2015 Quality management systems — Fundamentals and vocabulary https://www.iso.org/standard/45481.html). Quality indicators are used to benchmark the fulfilment of a requirement using a clearly defined numerator and denominator. Quality indicators are therefore always linked to a requirement and are a key part of a QA Scheme. In the ECIBC, integration of guideline development and QA proved challenging.

Thus, in 2017, the JRC started developing a methodological framework that integrates guideline recommendations and QA schemes. This framework will inform the new European Commission Initiative on Colorectal Cancer – ECICC) aimed at improving colorectal cancer quality of care.

The JRC invited a group of experts to develop this approach that required arriving at clear concepts and terminology for the QA field. This work begun with the updating of an existing systematic review (SR) on methods for guideline-based development of quality indicators [3]. The SR suggested that there is inconsistency in the way terminology and definitions are used in QA [3].

For example, a study by Becker et al. acknowledged that “there is no clear-cut definition of a quality indicator” [4]. According to Lawrence and Frede, a quality indicator is a “measurable element of practice performance for which there is evidence or consensus that it can be used to assess the quality, and hence change in the quality, of care provided” [5]. However, the same definition was used by Braithwaite et al. to describe performance indicators (PIs) [6]. In a review conducted in 2003 by the International Society for Quality in Health Care (ISQua) under contract to the World Health Organization describing structures and activities at national and international levels around the world to promote quality in health care, the terms “performance indicators”, “clinical indicators”, “indicators of quality of care”, “quality indicators” are reported as part of the various initiatives in different countries as measures for quality in healthcare [7]. The OECD defines its Health Care Quality Indicators (HCQI) as measures of health care quality that make use of readily available national hospital inpatient administrative data and other data sources [8]. The structure of the indicators based on hospital administrative data generally consists of definitions based on ICD-9-CM and ICD-10-WHO diagnosis and procedure codes. The National Institute for Health and Care Excellence (NICE) indicators generally measure outcomes that reflect the quality of care or processes linked by evidence to improved outcomes [9]. Outcomes are ideally, but not always, related to NICE quality standards that are used to focus on developing outcome measures that represent the overall quality of care in an area. The National Quality Forum refers to measurement systems as to how measures are used to achieve a goal [10]. Measurement systems vary by context, setting, and intended use. Measurement systems combine these aspects to make inferences about performance of a provider or a policy: the objective of the measurement system (cost or quality issue the system is trying to improve), the incentive mechanism the system will use to drive improvement, the risk-adjustment approach to standardize the population being measured in the system. A healthcare performance measure provides a way to calculate whether and how often the healthcare system does what it should. The specifications of a healthcare performance measure generally include the following key components: measure name and title; description (numerator and denominator definitions); target population; key terms, data elements, codes, and code systems used to define the target population; calculation algorithm; timing and time intervals; unit of accountability; data source(s); sampling and stratification method; risk adjustment method or exclusions. The Centers for Medicare and Medicaid define quality measures as tools that help us measure or quantify healthcare processes, outcomes, patient perceptions, and organizational structure and/or systems that are associated with the ability to provide high-quality health care and/or that relate to one or more quality goals for health care (https://www.cms.gov/medicare/quality-initiatives-patient-assessment-instruments/qualitymeasures/index.html). These goals include: effective, safe, efficient, patient-centered, equitable, and timely care. Measurement is a step in improving health care quality, and quality measures help drive that improvement through a consistent and accountable approach.

Our systematic review suggests that there is no comprehensive, well-defined conceptual framework for the integration of guideline development and QA [11]. For example, we found that there is no clearly accepted linkage of the QA terms and dimensions to the health questions that are answered by guidelines’ recommendations. The elements of a health question include population, interventions and comparisons, and the outcomes (PICO) that are the target of QA. However, none of the included studies evaluated the impact of guideline integrated quality indicator development on health outcomes [11]. The original systematic review included 14 method articles and 32 topic articles, the updated one (2010–2019) includes 17 new method articles and twice as many topic papers [3, 11]. This suggests that, although quality indicator development is a topic of high interest, there is minimal methodological advancement and the connection with guideline development methods is very limited.

Thus, the objective of this work is to propose a conceptual framework for guideline-based QA schemes building on clear definitions and questions asked in guidelines. This is the third in a series of three articles focusing on the topic at hand. The first article describes the approach to the work in general and the second article the updated systematic review [3, 11].

## Methods

The JRC convened a multidisciplinary group of 23 experts from key organizations representing the fields of guideline and QA development to identify potential challenges and propose solutions for an integrated methodological framework. The activity of the group was coordinated by a steering group composed of four researchers (HJS, TP, ML, EP), one from JRC and three from other organisations.

The work of the multidisciplinary group started with approximately 8 months of preparation for a workshop organised by the JRC in Ispra, Italy in June 2018. A description of the workshop methods, outcomes and expert participants is provided elsewhere [12]. This work was assessed in December 2017 by the Hamilton Integrated Research Ethics Board (HIREB) as a quality improvement study and exempt from full research ethics review. Participants provided verbal consent to participate in this workshop and surveys in accordance with the HIREB exemption review.

We carried out a mixed method approach to develop a conceptual framework relating health questions to QA through the use of consistent terminology. For each step we recorded the discussions and at least two participants took minutes; we also prepared summary documents of the discussions and circulated them to the whole group for further comments.

We utilized the results of the updated systematic review on methods for the guideline-based development of quality indicators [11] and we scanned the literature to identify terms and definitions used in this field. We prepared a first proposal for a 3-level conceptual framework applying existing terms for defining the underpinnings of quality indicators in relation to guideline PICO questions.

### Participants

A multidisciplinary group of experts in guideline or QA scheme methodology and development, or both, (including quality indicator and performance measures development) took part in the workshop and in subsequent virtual discussions. The group was composed by guideline and QA methodologists, IT technology specialists, epidemiologists, clinicians and a citizen advocate. In addition, the four members of the steering group and two JRC staff members participated. Twelve of these participants are in the ECIBC project [1].

### Mixed method approach

During the June 2018 workshop, the steering group presented the conceptual structure and drafts of definitions that were discussed both in small and large group sessions with the participants. We then compiled the outputs of the June 2018 workshop and circulated them to the entire group. We created a document with the terms and concepts and presented the revised conceptual framework during subsequent virtual meetings. Twenty-two members of the multidisciplinary group took part in the workshop, while one participated in the virtual meetings only.

### Final agreement

The comments and concerns collected during the workshop discussion were summarised and integrated in documents that were then circulated by email. Following the workshop, we held several rounds of discussion through email exchange and three teleconferences with the authors’ group, focused on the refinements of details and better presentation of the format of the framework. This part involved more informal discussions and final agreements. The steering group met regularly to iteratively integrate written comments and feedback from participants. We then obtained their final approval for the 3-level conceptual framework through email.

## Results

We created a 3-level conceptual framework for the definition of key elements for QA that linked them to the outcomes in the PICO health question approach for guidelines. To reduce confusion in the field, the framework requires common definitions of three key elements for QA: 1. quality indicators; 2. performance measures; 3. performance indicators.

Given the desired need for flexibility, we propose alternative terms for them that can be used interchangeably. Table 1 describes the definitions and concepts we developed.

**Table 1:** Definition of terms

**1. Quality indicators (may also be conceptualized as quality markers/quality constructs/quality parameters)**	
Purpose: to address specific aspects of the quality of healthcare [3].	
Definition: quality indicators are constructs that relate to structures, processes or outcomes of care, and are used to monitor, evaluate, and improve the quality of care. They can then be used to identify gaps in the quality of care, with potential for quality improvement. Quality indicators can be translated into performance measures allowing for rate-based measures (e.g., numerator and denominator) and can be utilized to assess the quality of screening, diagnosis, management, and/or care programmes, and, to develop tools to implement strategies for quality improvement. In the context of clinical guideline development, quality indicators should be based on people important outcomes selected for the healthcare questions included in the guidelines (health status indicators). Other indicators, sometimes (validated) surrogates, that represent population-based net benefits, e.g. existence of an organised screening programme or participation in screening programmes may also have to be established in order to guarantee that structures and processes are appropriate (structure and process indicators)	
**2. Performance measures (may also be conceptualized as performance tools or performance instruments)**	
Purpose: to quantify the performance of healthcare associated with a quality indicator.	
Definition: performance measures are tools or instruments to quantify or describe measurable elements of practice performance. Performance measures should attempt to measure performance that is directly attributable to a healthcare organization, a team or single actor, but not to independent environmental factors (that is, they avoid confounding by environmental factors).	
**3. Performance indicators (may also be conceptualized as performance estimates)**	
Purpose: to monitor aspects of health care performance associated to a quality indicator such as effectiveness, efficiency, and safety [13].	
Definition: performance indicators are quantifiable or measurable units or scores of practice. Performance indicators allow setting thresholds, ranges or other targets to permit comparisons and evaluations of practice performance that is directly attributable to a healthcare organisation, a team, or single actor.	

**Fig. 1:**
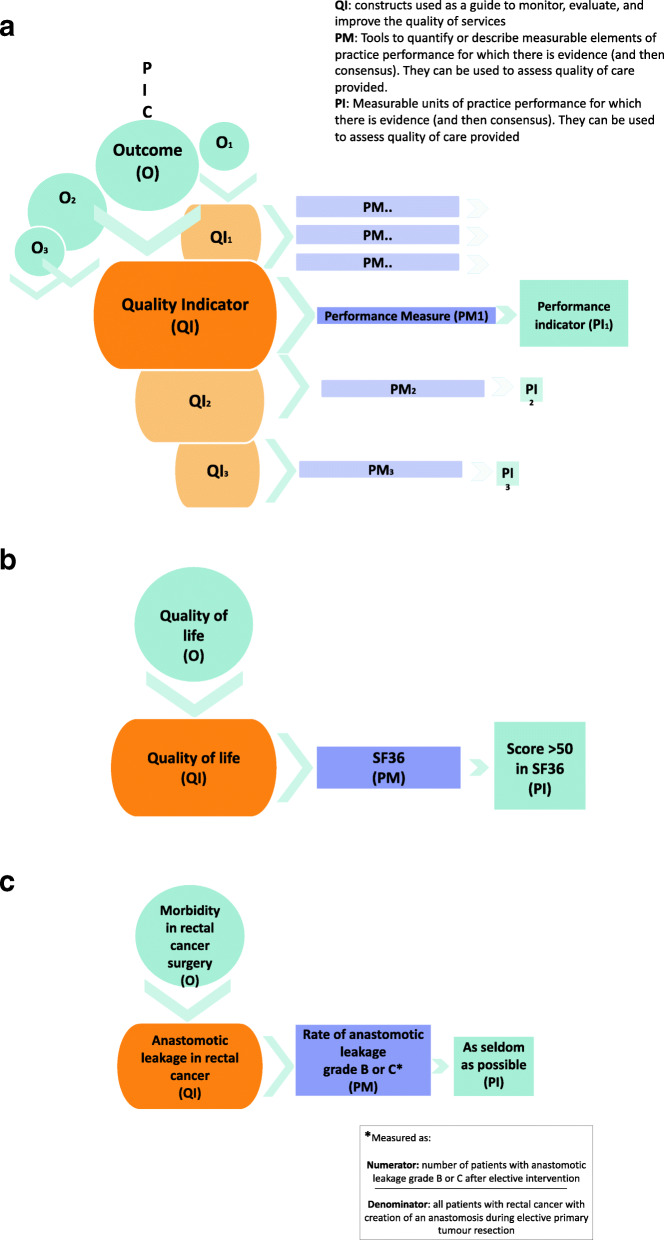
**a** describes the interconnectivity between the key elements and the PICO question as fundamental underpinning to the framework. The quality indicators should be directly linked to the health outcomes of a PICO question and the outcomes considered to generate a recommendation. **b** and **c** describe the three-layer approach from construct to tools or instruments to units of measurement based on two examples: quality of life and complications of therapy, leakage of an anastomosis, as health status indicators, respectively

During the three teleconferences following the workshop, we identified the need for refining the graphical presentation that connects the key elements and the PICO question as fundamental underpinnings to the framework (Fig. 1a). The quality indicators are typically directly linked to the health outcomes of a PICO question, hence of a recommendation, and the definition of performance measure is close to measuring outcomes. If structure or process indicators, as surrogates for health outcomes, are needed they should be supported by valid evidence that relates these indicators to the health outcomes of interest. Table 2 presents practical examples of application of the conceptual framework developed to health outcomes (Fig. 1b and c). Given the novelty of the approach, it is challenging to identify examples that could highlight the main features and possible challenges of our framework. However, the examples shown are those proposed during the teleconferences and through email exchanges. They are related to the direct experience of the group in guidelines development and QA methodology and exemplify alternative scenarios and applications of the framework.

**Table 2:** Examples of quality indicators, performance measures and performance indicators for different health questions. (*Note that the thresholds provided under performance indicator were contrived by the group for the purpose of these examples and not intended to represent an actual appropriate performance indicator for the topic)*

Quality Indicator [construct]	Performance Measure [rate-based measure of construct]	Performance Indicator [rate used to set threshold]
**Outcome: Quality of Life, e.g. for cancer care.**		
Quality of Life *(health status indicator)*	The Short Form (36) Health Survey (SF36)^1^	Scores above 50 on the SF36 functional domain
**Outcome: Complication of rectal cancer surgery**		
Anastomotic leakage in rectal cancer^2^ *(health status indicator)*	Rate of anastomotic leakage grade B or C (measured as: n° of pts. with anastomotic leakage grade B or C after elective interventions/ All pts. with rectal cancer with creation of an anastomosis during elective primary tumour resection)	As seldom as possible: Grade B or C anastomotic leakage following the creation of an anastomosis during surgery to treat rectal cancer
**Outcome: Mortality from colorectal cancer**		
Mortality from colorectal cancer *(health status indicator)*	Proportion of deceased people at time point x among all people diagnosed	< 20%
Participation in screening *(process indicator)*	Proportion of people, for whom screening is recommended, participating in screening among all the eligible people (e.g. governmental databases)	Higher than 95%
Existence of an organised screening programme *(structure indicator)*	Audit in which the requirements for a screening programme are assessed	100%
**Outcome: Venous thromboembolism (VTE) in hospitalized medical patients (deep venous thrombosis or pulmonary embolism)**		
VTE *(health status indicator)*	Proportion of hospitalized medical patients developing VTE over all the hospitalised patients	Less than 5%
Risk stratification for VTE in hospitalized patients *(process indicator)*	Proportion of eligible patients being risk stratified and treated according to risk stratification over all hospitalised patients eligible for risk stratification	100%
Clinical decision support system (CDSS) present *(structure indicator)*	Audit in which the availability of CDSS and policy for adoption of the use of CDSS are assessed.	100%

^1^ https://www.rand.org/health-care/surveys_tools/mos/36-item-short-form.html

^2^ German Guideline Program in Oncology (GGPO): Evidence-based guideline for colorectal cancer. Version 2.1 – January 2019

The three-layer approach from construct to tools or instruments to units of measurement is highlighted in these examples and allows for a bidirectional conceptualization that focuses on outcomes that matter to people.

In the examples, we distinguish between structure, process and outcome (from now on called “health status” to avoid confusion with the outcomes that are the starting point of our conceptual framework) indicators as proposed by the Donabedian model [13]. The health status indicators are directly linked to people important outcomes and refer to the effect of healthcare on the health status of the population. The process indicators refer to the delivery of care and the structure indicators refer to the context in which healthcare is delivered [13]. Process and structure indicators are usually linked to surrogate outcomes.

Quality indicators, classified as process, structure, outcome/health status, can be used to monitor, evaluate, and improve the quality of care; they can be measured though specific tools (performance measure), using defined thresholds (performance indicator).

## Discussion

We developed a conceptual framework that explains the role, meaning and interactions of the key elements when evaluating quality in healthcare, building on the questions asked in guidelines and answered through recommendations. In the context of developing an integrated guideline and QA framework for the European Commission, this work was triggered by inconsistency about the use of terms and definitions for key elements in QA.

We agreed on a 3-level structure connecting three key elements of QA: 1. quality indicators are constructs used as a guide to monitor, evaluate, and improve the quality of services; 2. performance measures are tools that quantify or describe measurable elements of practice performance; and 3. performance indicators are quantifiable and measurable units or scores of practice.

While finalizing the conceptual framework, we discussed the identification of quality indicators within an integrated methodology for guidelines and QA schemes. We agreed that identification should be based on a systematic and transparent approach [14]. Also, quality indicators that are not directly derived from specific guideline recommendations require special consideration and transparent reporting on the rationale for selecting.

In addition, quality indicators should satisfy important attributes that include being scientifically sound, based on evidence and strongly correlated with the quality of care provided [4]. Quality indicators should also be relevant to the selected problem and the field of application, feasible to identify and measure, and not susceptible to manipulation. We also suggested that quality indicators should be sensitive to change that means they should be able to capture possible changes in healthcare delivery [12].

Our work provides important advances in this field by clearly laying out the relation of the health question and the outcomes of interest to QA, its conceptual underpinnings and clarifying the terms used. Specifically, the contributions of this work to the literature include the identification of key elements in guideline-based QA and our examination of their relations and the development of a 3-level conceptual framework for their definition that linked them to the outcomes in the PICO health question approach for guidelines. Our conceptual framework contributes to bring clarity and consistency when referring to measuring quality in healthcare.

### Strengths and limitations

Strengths of this work are the involvement of a multidisciplinary group of experts in the field of guideline development and quality assurance and the structured process used to arrive to the final decision.

Table 1 shows that the conceptual framework proposed is suitable for different types of quality indicators related to different parts of the healthcare pathway like clinical, structural or process aspects. The graphical representation describes three key elements and how they are interconnected visually. This presentation should facilitate the uptake by others developing guideline-based quality indicators.

The group of experts participating in this process were selected, as a convenient sample, based on their expertise in guideline or QA scheme development, or both (including quality indicator and performance measures development), and in particular covering these relevant profiles: guideline and QA methodologists, IT technology specialists, epidemiologists, clinicians and a citizen advocate [12]. The experts involved are mainly coming from Europe and North America, but they are members of international organisations/networks (i.e. GRADE Working Group; Guidelines International Network; International Society for Quality in Health Care), so we think that their expertise and vision go beyond their places of origin/work. Since the work was related to the ECICC, the perspective taken was mainly linked to the European context, but we think and hope that the results of this work will go beyond Europe through processes of implementation and adaptation to different contexts.

One limitation of this work may be the lack of a systematic review specific to the terminology used to define quality indicators. However, our systematic review and the scoping work we did, supplemented by input from experts, made clear that there is confusing and overlapping definitions and concepts, requiring a consensus process to agree on terminology.

The possibility exists that other groups of experts working on the same topic may develop another approach. We believe, however, that the process we followed, including the previous experience with ECIBC, evaluation of existing methods and the involvement of a multidisciplinary group of experts, should increase generalisability and acceptance of our approach and reduce the likelihood of entirely different frameworks.

Formal application of the conceptual framework to guideline questions will be critical for future developments. The examples presented in Table 2 are related to the direct experience of the group in guidelines development and QA methodology. Furthermore, in the absence of any other validated framework, as shown in our systematic review, we will apply and evaluate our framework in the ECICC [7].

This and other future practical applications of the framework from different groups in different contexts may refine it based on its strengths and potential weaknesses.

### Implication for practice

The dissemination and implementation of the conceptual framework represents an opportunity to build on common concepts when evaluating quality in healthcare. The explicit interconnection between the health questions and the key elements of QA should facilitate and encourage the collaboration between groups involved in the development of evidence-based guidelines and QA schemes.

### Implication for research

Future application of this conceptual framework in different contexts by different groups involved in QA is key to strengthening the proposed framework, and to refining the definitions as well as the graphical representation. They should be evaluated for their utility and ability to provide clarity and to help in the development of guideline-based QA schemes.

## Conclusions

Our conceptual framework provides clarity and consistency when referring to measuring quality in healthcare, through an evidence review and consensus approach by experts in this field.

Together with the key themes identified in the consultation process with experts [12], it will be a roadmap to inform the future work on the development of a methodological framework for a guideline-based QA scheme and, in the meantime, it serves as considerations for practical guideline and QA development groups.

These findings will be applied in the forthcoming ECICC and for the future updates of ECIBC. These are large-scale integrated projects aimed at improving healthcare quality across Europe through the development of guideline-based QA schemes; this will help in implementing and improving our approach.

## Data Availability

Not applicable.

## Abbreviations

ECIBC: European Commission Initiative on Breast Cancer
ECICC: European Commission Initiative on Colorectal Cancer
HCQI: Health Care Quality Indicators
ISQua: International Society for Quality in Healthcare
JRC: Joint Research Centre
NICE: The National Institute for Health and Care Excellence
PICO: Population, Intervention, Comparison, Outcome
SR: Systematic review
QA: Quality Assurance
